# Hunger improves reinforcement-driven but not planned action

**DOI:** 10.3758/s13415-021-00921-w

**Published:** 2021-10-15

**Authors:** Maaike M.H. van Swieten, Rafal Bogacz, Sanjay G. Manohar

**Affiliations:** Nuffield Department of Clinical Neuroscience, University of Oxford, Oxford, UK

**Keywords:** Motivation, Learning, Decision-making, Reward, Planning

## Abstract

Human decisions can be reflexive or planned, being governed respectively by model-free and model-based learning systems. These two systems might differ in their responsiveness to our needs. Hunger drives us to specifically seek food rewards, but here we ask whether it might have more general effects on these two decision systems. On one hand, the model-based system is often considered flexible and context-sensitive, and might therefore be modulated by metabolic needs. On the other hand, the model-free system’s primitive reinforcement mechanisms may have closer ties to biological drives. Here, we tested participants on a well-established two-stage sequential decision-making task that dissociates the contribution of model-based and model-free control. Hunger enhanced overall performance by increasing model-free control, without affecting model-based control. These results demonstrate a generalized effect of hunger on decision-making that enhances reliance on primitive reinforcement learning, which in some situations translates into adaptive benefits.

**Supplementary Information:**

The online version contains supplementary material available at 10.3758/s13415-021-00921-w.

## Significance statement

The prevalence of obesity and eating disorders is steadily increasing. To counteract problems related to eating, people need to make rational decisions. However, appetite may switch us to a different decision mode, making it harder to achieve long-term goals. Here we show that planned and reinforcement-driven actions are differentially sensitive to hunger. Hunger specifically affected reinforcement-driven actions, and did not affect the planning of actions. Our data shows that people behave differently when they are hungry. We also provide a computational model of how the behavioral changes might arise.

## Introduction

Hunger is an adaptive motivational state that drives us to eat, restoring homeostatic balance (Saper et al., 2002). However, maladaptive behavior in the context of hunger generates many clinical problems. One in seven adults suffers from obesity, one of the most serious health issues in the developed world, and 10% suffer from a range of eating disorders. One critical contributing factor may be the changes in decision-making produced by hunger.

One well-studied effect of hunger is to amplify the value of reward, both specifically for food (Epstein, Truesdale, Wojcik, Paluch, & Raynor, 2003; Malik, McGlone, Bedrossian, & Dagher, 2008), and even for generic reward signals (Haase, Cerf-Ducastel, & Murphy, 2009; Siep et al., 2009; Aitken, Greenfield, & Wassum, 2016; Cone et al., 2016; Papageorgiou, Baudonnat, Cucca, & Walton, 2016)(Simon et al., 2017; Briers, Pandelaere, Dewitte, & Warlop, 2006). This modulation of value may feed into primitive reinforcement systems (Daw and O’Doherty in Glimcher and Fehr (2013)), strengthening simple action learning. This kind of value-based action reinforcement is inflexible, and is more strongly engaged in people with eating disorders (Voon et al., 2015). However, to allow for flexible, context-sensitive action selection, these primitive systems must be guided by a planning system, which computes *how* to get to a reward using a causal model of the world, rather than simply engaging actions that previously led to reward. The planning system is sensitive to metabolic needs insofar as they determine our goals (Dickinson, 1985; Dickinson & Balleine, 1994; Aw et al., 2009; Friedel et al., 2014), but it is not known whether needs can make us generally better or worse at goal-directed planning.

In this study, we asked whether hunger promotes primitive decisions based on reinforcement, or planned actions. Simple reinforcement is model-free, with memory only about how rewarding actions were in particular states. In contrast, the model-based planning system, uses knowledge about how actions change the state of the world. Would both of these systems be sensitive to biological needs?

On one hand, we might expect the model-free system to be modulated by hunger, because it may rely more on primitive neural systems. Circulating hormones that signal metabolic need target subcortical brain areas that regulate feeding (Zigman, Jones, Lee, Saper, & Elmquist, 2006; Elmquist, Bjørbæk, Ahima, Flier, & Saper, 1998). In particular, leptin inhibits and ghrelin activates dopaminergic neurons in the ventral tegmental area, and could therefore modulate learning and decision-making via the mesolimbic pathway (Hommel et al., 2006; Figlewicz, MacDonald Naleid, & Sipols, 2007; Abizaid et al., 2006). This automatic mechanism could presumably be advantageous, since hunger may trigger reward-driven behavior enabling organisms to adapt to the variability in environmental abundance (de Ridder, Kroese, Adriaanse, & Evers, 2014; Symmonds, Emmanuel, Drew, Batterham, & Dolan, 2010; Levy, Thavikulwat, & Glimcher, 2013; Shabat-Simon, Shuster, Sela, & Levy, 2018).

On the other hand, we might expect a higher-level, model-based system to be modulated by hunger. It is by definition more flexible, being sensitive to state and context. Higher-level control may be decreased by hunger. For example, hunger promotes impulsivity, preventing us from reaching long-term goals (Bartholdy et al., 2016; Kirk & Logue, 1997; Skrynka & Vincent, 2019). Cognitive control is regulated by homeostatic hormones (Higgs et al., 2017), and is impaired in obesity (Hassenstab et al., 2012) but enhanced in anorexia nervosa (Compan, Walsh, Kaye, & Geliebter, 2015).

**Fig. 1:**
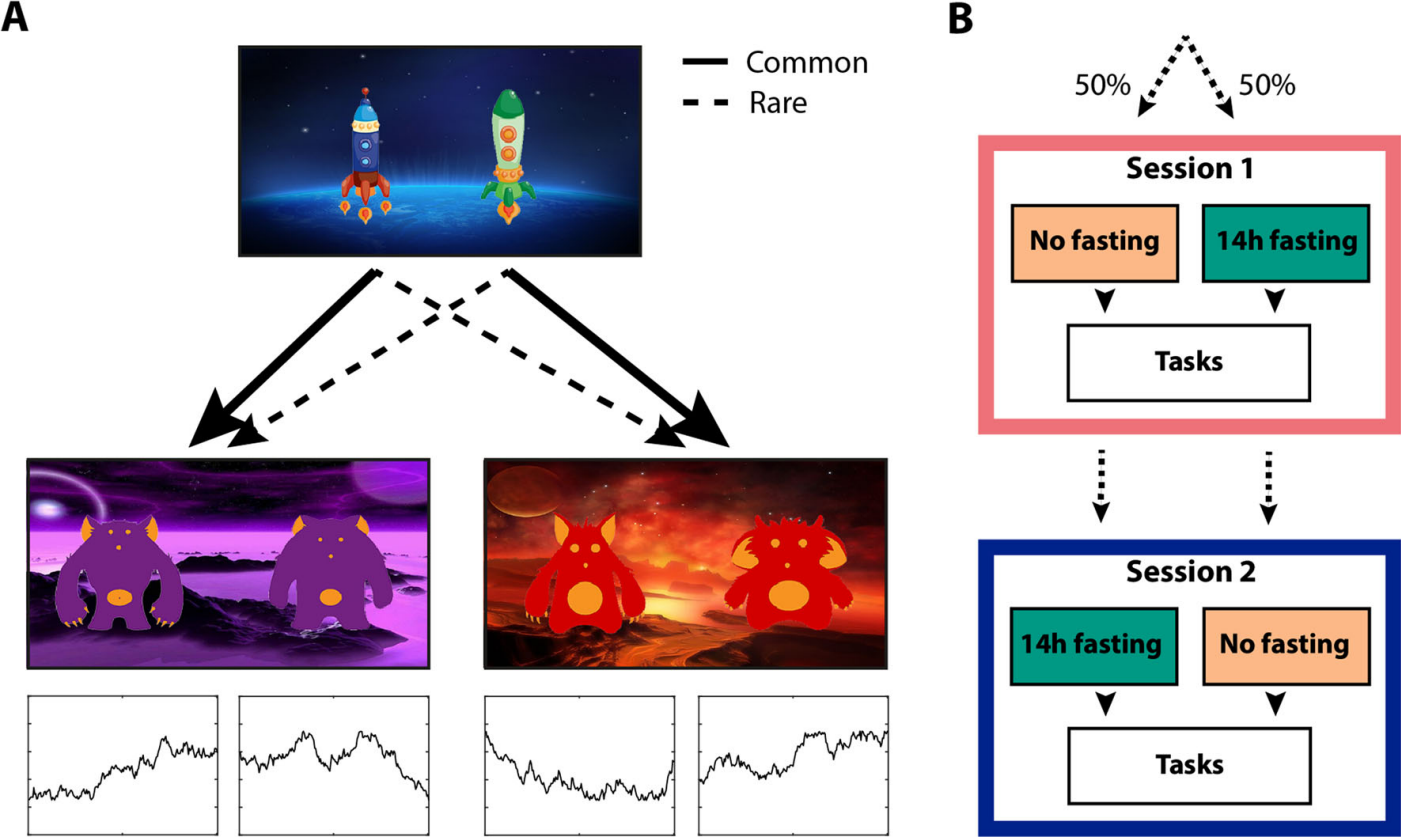
Study design. **A)** Schematic of the task design developed by (Daw et al., 2011). Each first-stage choice rocket flew predominantly to one of the second-stage planets (common transition: 70% of the trials) and sometimes to the other second-stage planet (rare transition: 30% of the trials). Each second-stage alien probabilistically led to a reward. The reward probabilities for each second-stage alien fluctuated across trials between 25% and 75% according to a Gaussian random walk. Task instructions and images were obtained from Kool, Cushman, and Gershman (2016). **B)** Participants were tested in a within-subjects counterbalanced, randomized crossover design. Participants were tested on two separate days approximately 1 week apart. One session took place after 14 h of fasting, the other session after consuming a full meal

In the present study, we employed a two-stage decision-making task (Daw, Gershman, Seymour, Dayan, & Dolan, 2011) to examine if hunger modulated the model-free or model-based decision system. In this task, people must choose between two rockets, each going predominantly to one of the second-stage planets (Fig. 1A). Once on a planet, they choose between two aliens that probabilistically deliver a reward. If a reward is obtained, it pays off to return to that planet on the next trial by selecting the same first-stage rocket again. Crucially, the rockets occasionally go to the other planet (a “rare transition”). If a reward is earned in this situation, it pays off to choose the *other* rocket next time, because that rocket is more likely to bring you back to the same second-stage planet. The simple, “model-free” approach is to repeat the rocket choice whenever it was rewarded. A more sophisticated, “model-based” strategy is to consider that rockets do not always go to the same planet and plan ahead to reach the most rewarding planet.

We showed that increased metabolic need enhanced reflexive model-free control of behavior, without affecting deliberate model-based control. Although food deprivation has been shown to increase impulsivity, this study provides evidence that relying on a reflexive model-free system does not make hungry people maladaptive, when the deliberate model-based system remains unaffected.

## Methods

### Participants

Thirty-two healthy volunteers (females: 20, mean age: 25.6 ± 6.5) were recruited for this study. All participants were healthy, had no history of psychiatric diagnoses, neurological or metabolic illnesses, and had not used recreational drugs in the past 3 months. All participants had a normal weight (body mass index: 22.9 ± 3.2 kg/m^2^), regular eating patterns and no history of eating disorders. Each participant gave written informed consent, and the study was conducted in accordance with the guidelines of the University of Oxford ethics committee. We estimated a medium effect size (Cohen’s *d* = 0.50) from three published within-subjects studies using a similar sequential decision-making task. We assume that an effect of hunger used in the current study lies in a similar range as compared to the interventions used in these studies (a pharmacological manipulation with L-DOPA (Wunderlich, Smittenaar, & Dolan, 2012), a transcranial magnetic stimulation intervention (Smittenaar et al., 2013), psychosocial stress intervention (Radenbach et al., 2015)). We then used G*Power (3.1.9.7) software and estimated that we need 32 participants to obtain a power of 0.80, at *α* = 0.05. Our observed power (with an effect size of 0.44) is 0.78. All data and code are openly available at https://data.mrc.ox.ac.uk/data-set/effects-hunger-model-based-and-model-free-decision-making (van Swieten, Manohar, & Bogacz, 2021).

### Manipulation of metabolic state

Participants were tested in a within-subjects counterbalanced, randomized crossover design for the effects of food deprivation on planning (Fig. 1B). Sessions were approximately 1 week apart (at least 4 days, but no more than 14 days). All sessions took place at the same time of day between 10:00 am and 1:00 pm, to minimize time-of-day effects. For one session, participants were asked to refrain from eating and drinking caloric drinks from 8:00 pm the night prior to testing. For the other session, participants were asked to eat normally the day before and consume a full breakfast within 1 h of arriving at the lab for testing. We assessed the effect of food deprivation on self-reported feelings of hunger and mental state using a computerized Visual Analogue Scale (Bond & Lader, 1974; Flint et al., 2000). Participants were asked to place a cursor on a 100-mm scale with positive or negative text ratings anchored at either end. This assessment provided a subjective measure of whether the manipulation worked. Participants also performed a risk-taking, attention and learning task not described in this paper.

### Paradigm

In the two-step task, participants make a series of choices between two stimuli, which lead probabilistically to one of two second-stage states (Fig. 1A). Each first-stage rocket leads more frequently (70%) to one of the second-stage states (a “common” transition), and in a minority of the choices (30%) to the other second-stage state (a “rare” transition). These second-stage states require a choice between two aliens that offer different probabilities of obtaining a monetary reward. To encourage learning, the reward probabilities for each second-stage alien fluctuated across trials between 25% and 75% according to a Gaussian random walk (Daw et al., 2011).

To solve this task, a model-free strategy would involve choosing the same rocket when it previously resulted in a reward. This would occur irrespective of whether the transition to the second-stage was common or rare (i.e., whether the planet was expected or unexpected), because the model-free system is insensitive to the structure of state transitions within the task. In contrast, a model-based strategy would necessitate *switching* to the other rocket after a reward, if the transition was a rare one (i.e., if the chosen rocket went to the unexpected planet). This is because participants can use their model of which rocket tends to lead to which planet, to maximize their chances of obtaining future rewards.

The task consisted of 201 trials, divided into three blocks of 67 trials separated by short breaks. If participants failed to enter a choice for a first or second-stage choice within 2 s, the trial was aborted and not included in further analyses. A new set of randomly drifting reward distributions was generated for each participant. Participants received 1 pence for every point they earned.

Before completing the full task, participants were extensively trained on different aspects of the task. Participants sampled 25 times from aliens with different reward probabilities. They were also told that each rocket preferentially goes to one of the planets, but they were not instructed on which rocket goes to what planet or the explicit transition probabilities (Kool, Cushman, & Gershman, 2016). Finally, participants practiced the full task for 25 trials. There was no response deadline for any of the sections of the training phase. Different rockets, planet colors, and aliens were used for the training and experimental phase, and these were counterbalanced across conditions and participants.

### Stay-switch behavior

The one-trial-back stay-switch analysis is the most widely used method for characterizing behavior on the two-step task (Daw et al., 2011; Akam, Costa, & Dayan, 2015; Wunderlich et al., 2012). This method quantifies the tendency of a participant to repeat the choice made on the last trial or switch to the other choice, as a function of the outcome and transition on the previous trial. We considered four possible outcomes: Common-Rewarded (*CR*), Rare-Rewarded (*RR*), Common-Unrewarded (*CU*), and Rare-Unrewarded (*RU*). Model-based and model-free indices were computed from the stay probabilities following each outcome according to:

1 $$ \begin{array}{@{}rcl@{}} \text{MF} &=& (P(\text{stay}|CR) + P(\text{stay}|RR)) - (P(\text{stay}|CU) \\ &&+ P(\text{stay}|RU)), \end{array} $$

2 $$ \begin{array}{@{}rcl@{}} \text{MB} &=& (P(\text{stay}|CR) + P(\text{stay}|RU)) - (P(\text{stay}|CU) \\ &&+ P(\text{stay}|RR)). \end{array} $$

We also examined whether hunger modulated other measures of simple reinforcement learning. We found no effect of hunger on changes in model-free control for second-stage choices or for action-specific, stimulus-irrelevant choices at the first-stage (Fig. S1).

Statistical analyses were implemented in MATLAB and SPSS (IBM Corp. Released 2019. IBM SPSS statistics for Windows, Version 26.0. Armonk, NY: IBM Corp.). We report the effect size with Cohen’s *d* and ${\eta _{p}^{2}}$ and report the 95% confidence interval (CI) of the difference between groups.

### Computational modeling

We used a hybrid computational model that assumes that the model-based and model-free systems both contribute to choice behavior (Daw et al., 2011). Model-based and model-free algorithms learn the value of the stimuli that appear in the task in three different pairs. There is one first-stage pair (*s*1 ∈{1,2}) and two second-stage pairs consisting of two stimuli each (*s*2 ∈{3,4,5,6}). The indices *s*1 and *s*2 refer to stage 1 and stage 2, respectively. The index *t* refers to the trial number.

At the first-stage, model-free ‘cached’ values were updated using a temporal difference algorithm. This algorithm learns to maximize the total outcome by strengthening or weakening associations between the first-stage state and the first-stage actions, depending on whether a reward followed that action or not:

3 $$ \begin{array}{@{}rcl@{}} Q_{s1}^{\text{MF}}(t+1) &=& Q_{s1}^{\text{MF}}(t) + \alpha_{1} (Q_{s2}(t) - Q_{s1}^{\text{MF}}(t)) \\&&+ \alpha_{21} (r - Q_{s2}(t)), \end{array} $$

where *α*_1_ is the learning rate for the first-stage. The parameter *α*_21_ determines the extent to which the second-stage reward prediction error influences first-stage choices (which is equivalent to the temporal discounting term *α*_1_ × *λ* in the model by Daw, Gershman, Seymour, Dayan, and Dolan (2011)).

Model-based values were computed for each first-stage stimulus and every trial in a forward-looking manner by multiplying the state value of the best second-stage option with the state transition probabilities:

4 $$ \begin{array}{@{}rcl@{}} Q_{1}^{\text{MB}} &=& 0.7 \times \max(Q_{s2,3}, Q_{s2,4}) + 0.3\\ &&\times \max(Q_{s2,5}, Q_{s2,6}), \end{array} $$

5 $$ \begin{array}{@{}rcl@{}} Q_{2}^{\text{MB}} &=& 0.3 \times \max(Q_{s2,3}, Q_{s2,4}) + 0.7 \\&&\times \max(Q_{s2,5}, Q_{s2,6}). \end{array} $$

Model-based learning was simplified and the transition probabilities were not updated by explicitly modeling state prediction errors. Simulations by the authors of the original task showed that learning of state transitions quickly converge to stable values (see Supplementary Materials of Daw et al., (2011)).

The hybrid model computes the actual value that is used in determining first-stage choices as a weighted combination of the model-based (*Q*_MB_) and model-free (*Q*_MF_) values. The first stage Q values are computed the following way:

6 $$ Q_{s1}^{\text{hybrid}} = \beta_{\text{MB}} Q_{s1}^{\text{MB}} + \beta_{\text{MF}} Q_{s1}^{\text{MF}}, $$

where *β*_MB_ and *β*_MF_ are the weighting parameters for model-based and model-free control, respectively (and are equivalent to *β*_1_ × *ω* and *β*_1_ × (1 − *ω*) in the model by Daw et al., (2011)). Note that in a pure model-free approach *β*_MB_ = 0, and in a pure model-based approach *β*_MF_ = 0.

Q-values for the four second-stage stimuli were updated according to the reward prediction error (Rummery and Niranjan, 1994):

7 $$ \begin{array}{@{}rcl@{}} Q_{s2}(t+1) = Q_{s2}(t) + \alpha_{2} (r - Q_{s2}(t)), \end{array} $$

where *α*_2_ is the learning rate for the second-stage.

A first-stage choice depends on the relative difference in stimulus values between *Q*_*s*1,1_ and *Q*_*s*1,2_ and the choice *C* on the previous trial, which takes on the value 1 when the current choice equals the previous choice. The parameter *π* captures first-stage choice perseverance. Using the softmax choice function, the probability of choosing a first-stage stimulus was computed according to:

8 $$ P_{1} = 1/ (1+ \exp(- (Q_{s1,1}^{\text{hybrid}} - Q_{s1,2}^{\text{hybrid}}) - \pi C )), $$

and for the second-stage:

9 $$ P_{3} = 1/ (1+ \exp(-\beta_{2} (Q_{s2,3} - Q_{s2,4})), $$

where *β*_2_ control the randomness of second-stage choices.

We used a hierarchical model-fitting strategy that takes into account the likelihood of individual participant choices given the individual participant parameters and also the likelihood of the individual participant parameters given the parameter distribution in the overall population across conditions. This two-stage hierarchical procedure is an estimation strategy of the iterative expectation-maximization algorithm (EM) (MacKay, 2003; Guitart-Masip et al., 2011). This regularizes individual participants’ parameter fits, rendering them more robust toward over-fitting. To infer the maximum-a-posteriori estimate of each parameter for each participant, we set the prior distribution to the maximum-likelihood given the data of all participants and then used EM for the two conditions separately to obtain parameter estimates for each condition. The statistical significance was tested using paired *t* tests with respect to the Gaussian scaled model parameters (see Supplementary Material for the transformation of parameters). The reported *p* values were corrected for multiple comparisons using the Bonferroni method.

## Results

As expected, participants rated their subjective feelings of hunger significantly higher after 14 h of fasting than after eating a full meal (Wilcoxon signed-rank test: [*Z* = − 4.84, *p* < 0.0001, *d* = 0.86]), indicating that the manipulation was successful.

We first examined the influence of food deprivation on choice behavior. During each session, a participant undertook 201 trials, of which on average 2.3 ±s.d. 3.1 trials were not completed due to failure to enter a response within the 2 s time limit. Food deprivation did not affect the number of missed trials [*t*_31_ = 1.19, *p* = 0.245, *d* = 0.22, 95% CI [-0.61 2.29]] or median response times for first- and second-stage choices (first-stage RT_hungry_ = 616 ms, RT_sated_ = 640 ms; paired *t* test, [*t*_31_ = − 1.29, *p* = 0.207, *d* = 0.23, 95% CI [-55.10 12.41]], second-stage RT_hungry_ = 788 ms, RT_sated_ = 821 ms; paired *t* test, [*t*_31_ = − 1.25, *p* = 0.220, *d* = 0.22, 95% CI [-88.65 21.34]]), suggesting that food deprivation did not alter overall attention in this task.

### Food deprivation only modulated model-free control

**Fig. 2:**
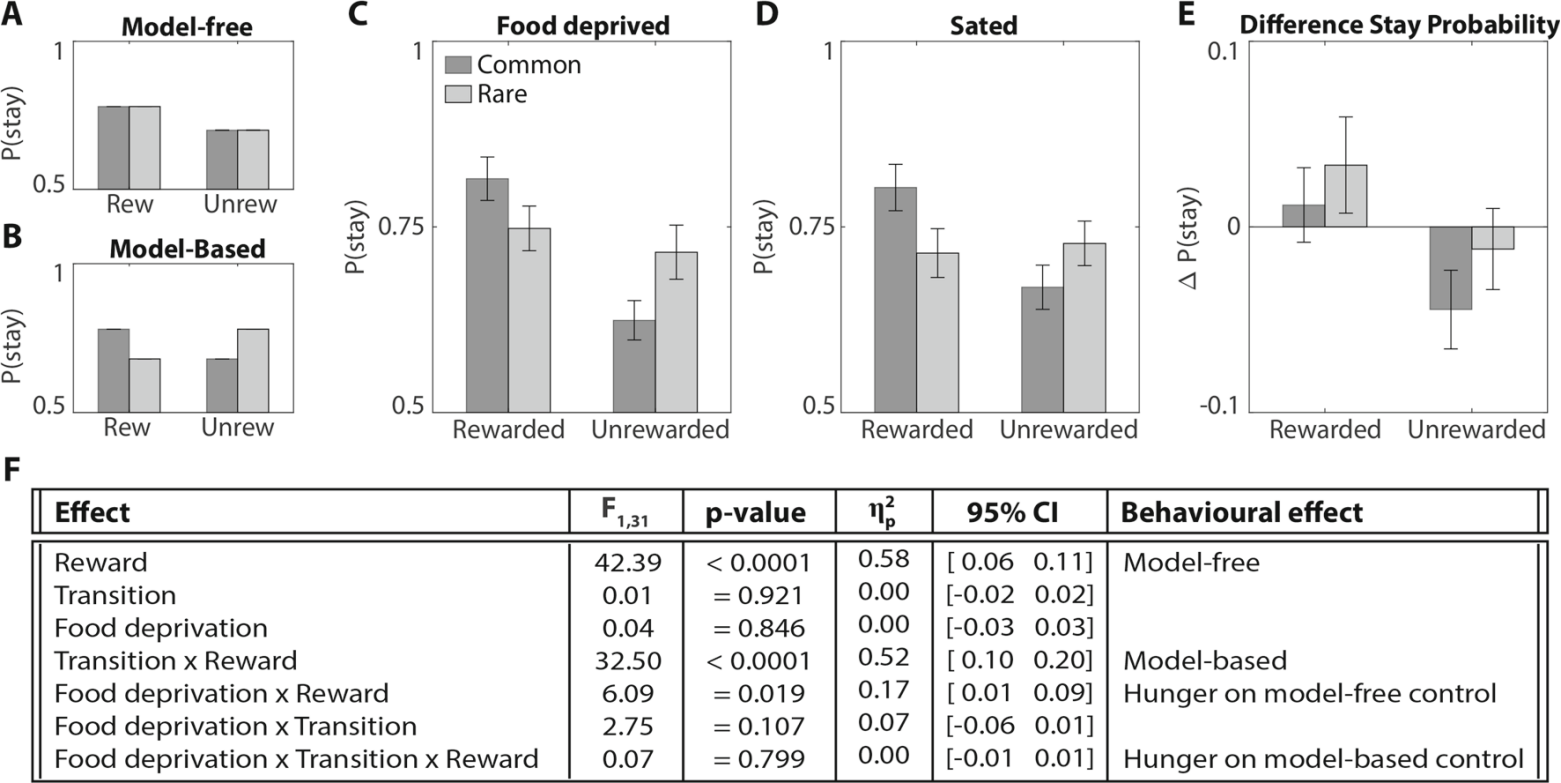
Stay-Switch behavior at first-stage. Model-based and model-free strategies for reinforcement learning predict different patterns by which outcomes experienced after the second-stage impact first-stage choices on subsequent trials (Daw et al., 2011). **A)** Simulation of choice behavior driven by the model-free system. Rewards increase the likelihood of choosing the same stimulus on the next trial, regardless of whether that reward occurred after a common or rare transition. **B)** Simulation of choice behavior driven by the model-based system, which relies on an interaction between transition type and reward outcome. **C)** Experimental data of food deprived participants. **D)** Experimental data of sated participants. **E)** Change in stay probability with food deprivation. Food deprivation increased the tendency to stay after receiving a reward and increased the tendency to switch after reward omission. *Error bars* represent SEM. **F)** Statistics stay behavior: three-way repeated measures ANOVA

To dissociate model-based and model-free control, we measured the tendency to “stay” with the same first-stage choice as the previous trial. Model-free and model-based strategies predict distinct patterns of stay behavior. A model-free reinforcement learning strategy predicts actions repeat when reinforced, i.e., a main effect of reward (Fig. 2A), whereas a model-based learning strategy predicts a crossover interaction between reward outcome on the second-stage and the type of transition (Fig. 2B). This arises because model-free and model-based strategies predict opposing stay probabilities on trials following a rare transition. After a rare transition, the model-free system updates the value of the first-stage *chosen* stimulus, such that reward promotes staying with the current choice. The model-based system instead updates state values, and so rewards after a rare transition will promote switching, since the *unchosen* first-stage stimulus is more likely to lead to the previously rewarded second-stage state.

We examined whether the probability of staying or switching depended on the level of food deprivation (hungry or sated), the transition type (common or rare), and the reward on previous trial (reward or no reward), using a three-way repeated measures ANOVA (Fig. 2C–F). Participants used both model-free and model-based strategies to solve this task, which is consistent with previous studies (Daw et al., 2011; Wunderlich et al., 2012; Deserno et al., 2015).

The key analyses concerned whether hunger modulated these learning strategies (Fig. 2F). Food deprivation increased the tendency to repeat the same choice after receiving a reward, but not after reward omission, showing that food-deprived individuals relied more on model-free strategies. Hunger did not affect overall stay behavior or model-based strategies.

Demographic factors may potentially modulate the effect. Including age as a covariate had no effect on the results. Including a median split of body mass index (BMI) showed possible inter-individual differences, with strong hunger effects on model-free learning in higher BMI individuals (see Supplementary Material) but not in lower BMI individuals. However, we were not powered to detect between-subject effects, and we do not interpret these further.

**Fig. 3:**
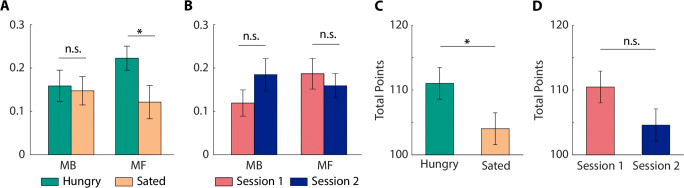
Hunger increased model-free control and performance. **A)** Food deprivation increased the relative contribution of the model-free (MF), but not model-based (MB), system to stay behavior at stage 1. **B)** Training did not significantly alter the contribution of the MB or MF system to choice behavior. **C)** Food deprivation increased the number of points earned. **D)** Session order had no significant impact on the number of points earned. *Error bars* represent SEM. *p < 0.05

The contributions of model-free and model-based strategies to choice behavior can be summarized with an alternative index (Eqs. 1 and 2). The current metabolic state modulated the model-free (MF) index [*t*_31_ = 2.47, *p* = 0.019, *d* = 0.44, 95% CI [0.02 0.19]], but not the model-based (MB) index ([*t*_31_ = 0.26, *p* = 0.799, *d* = 0.05, 95% CI [-0.08 0.10]]; Fig. 3A). The session order and the amount of training did not alter MB or MF indices ([*p* > 0.2]; Fig. 3B). These analyses confirmed that the manipulation of metabolic state, rather than training, caused the effects observed in this study. This observation corresponds with earlier reports that extensive training did not alter the trade-off between the model-based and model-free system (Grosskurth et al., 2019).

### Food deprivation enhanced performance

Although some studies have reported a significant correlation between overall performance and enhanced model-based control (Wunderlich et al., 2012), recent studies have shown that the number of points may not be strongly correlated to either model-based or model-free control (Akam et al., 2015; Kool et al., 2016). In this study, food deprivation enhanced performance, measured as the number of points earned in the task ([*t*_31_ = 2.25, *p* = 0.032, *d* = 0.40, 95% CI [0.64 13.36]]; Fig. 3C). The total number of points earned did not differ between the first and second testing days ([*t*_31_ = 1.84, *p* = 0.075, *d* = 0.32, 95% CI [-0.63 12.38]]; Fig. 3D), suggesting that more experience with the task structure did not improve performance (Grosskurth et al., 2019).

### Computational modeling confirmed model-free effects

**Fig. 4:**
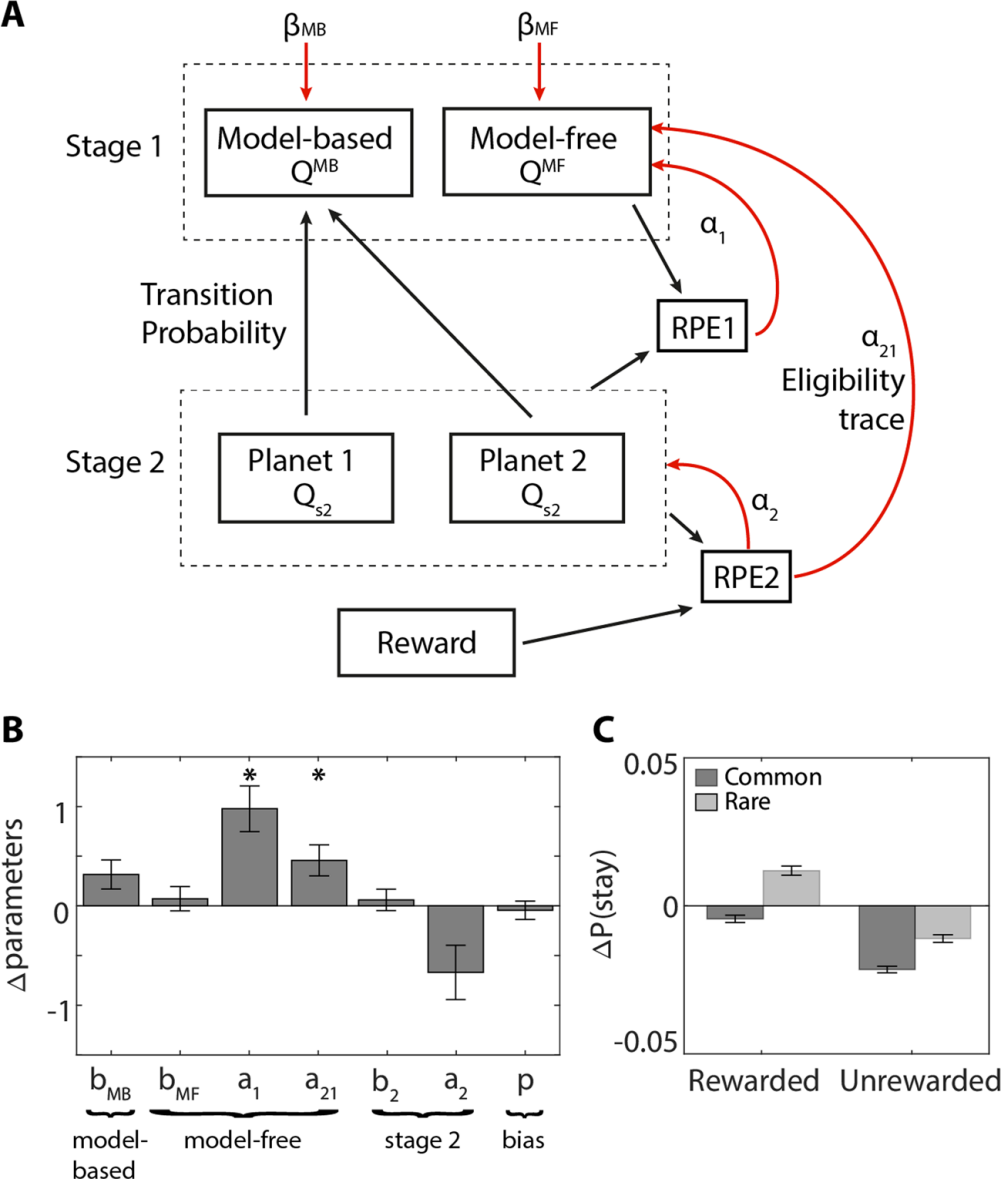
Computational modeling results. **A)** First-stage choices depend on a model-based and model-free component. Model-based values are computed by multiplying the stimulus with the highest value of both second-stage planets with their respective transition probabilities. Model-free values are updated using a first-stage reward prediction error (RPE1; scaled by *α*_1_), which captures the difference between the value of the chosen option in the first-stage and the chosen option in the second-stage (state transition), and an eligibility trace of the second-stage reward prediction error (RPE2). RPE2 captures the difference between the reward received and the value of the chosen second-stage option (scaled by *α*_2_). The model assumes that the second-stage prediction error influences the first stage values with a learning rate *α*_21_, because it assumes that first-stage choice leaves eligibility trace that makes *Q*^MF^ modifiable according to subsequent prediction errors. *Black arrows* indicate contribution to. *Red arrows* indicate that values are scaled by. **B)** Differences in Gaussian scaled parameters between food-deprived and sated conditions. Positive values indicate that the parameter estimate was higher when food-deprived compared to sated (food deprived > sated). **C)** Surrogate data simulated with the original model using best-fitting parameters for this model (Table S1) revealed the key pattern in stay behavior as shown by the experimental data in Fig. 2E. *Error bars* represent SEM. * *p* < 0.05

The behavioral pattern in Fig. 2E deviates from the pattern we expected from a pure model-free effect. Some studies have also criticized the interpretation of the stay-switch measures, in Eqs. 1 and 2, as mapping onto model-free and model-based systems, respectively (Grosskurth et al., 2019). Furthermore, model-based behavior is sometimes confused as model-free behavior (Feher da Silva & Hare, 2020). Therefore, we corroborate the behavioral results with computational modeling to assess the effects of food deprivation, and attribute these effects to a specific computational process. The complexity of the task and the contribution of model-based and model-free strategies to choice behavior were captured by a seven parameter model (Fig. 4A). These seven parameters can be grouped into four categories based on their functional roles: the model-based system, model-free system, second stage or bias. The quality of the fitting procedure was verified with a parameter recovery analysis (see Supplementary Material). All parameters were well recovered (0.65 ≤ *R* < 0.95) and the model fitting procedure did not introduce spurious correlations between the other parameters (|*R*| < 0.4; Fig. S2).

We found that food deprivation significantly increased the learning rate for first-stage choices [*α*_1_, *p* < 0.001, *d* = 0.76, 95% CI [0.51 1.45]] and the contribution of second-stage prediction errors [*α*_21_, *p* = 0.042, *d* = 0.52, 95% CI [0.14 0.78]]. These data suggest that hunger increased model-free learning. Hunger did not affect any of the other parameters ([*p* > 0.14]; Fig. 4B). Surrogate data generated with the best fitted parameters (Table S1) confirmed that the key difference in stay behavior (Fig. 2E) was captured by the estimated parameters (Fig. 4C). The modeling results confirmed that hunger increased model-free learning, without affecting model-based control, which explains the pattern observed in Fig. 2E.

## Discussion

Truly adaptive behavior involves changing policy in response to not only outcome contingencies, but also our metabolic state. In this study, we used a sequential decision-making task to test whether hunger modulates simple reflexive decisions or prospectively planned actions. Hunger enhanced overall performance by increasing model-free control, without changing model-based control. These results indicate that hunger enhances adaptive behavior by boosting reinforcement learning, without affecting cognitive processes that are required for future planning.

To solve the two-step task, people can either use model-free learning and simply repeat actions that yielded rewards, or they can use model-based learning to track *states* that yielded rewards, and plan actions to reach those states. These two strategies entail very different computations. These may in turn rely upon different brain systems (Lee, Shimojo, & O’Doherty, 2014) that could respond differently to resource depletion or repletion. The sequential task allowed us to track both of these modes of decision-making. In line with previous studies (Daw et al., 2011; Wunderlich et al., 2012; Grosskurth et al., 2019), our participants used a mixture of model-based and model-free strategies to solve the task.

Hunger increased the importance of model-free control, characterized by a main effect of reward and increased learning rates derived from computational modeling. At first glance, it might be surprising that hunger increased both *α*_1_ and *α*_21_. The exact parameterization of the model may matter due to the hierarchical nature of the fitting procedure, which assumes that each parameter has a similar distribution across participants. A previous model of the task formulated *α*_21_ as split into the product of the stage 1 learning rate *α*_1_ and an eligibility parameter *λ*. In that formulation, there is a trade-off between *α*_1_ and *λ*. Applied to our data, that formulation showed worse recoverability of the eligibility trace parameter (van Swieten (2020) Fig. 7.5). Our re-parameterized model is algebraically equivalent to previous models (e.g., Daw et al., (2011); Grosskurth et al., (2019)), but may give a clearer separation between the two steps of learning. It is important to establish recovery of the models (Palminteri, Lefebvre, Kilford, & Blakemore, 2017) and their validity (Wilson & Collins, 2019). We were able to recover parameters well in our simulations (Figs. S2 & S3). Moreover, we performed a model comparison by including hunger as a factor, or excluding it. We find significantly better model fits with hunger included as a factor (ΔAIC = 41), in line with both the behavioral results, and the significant difference in the recovered parameters.

When food deprived, participants won more points, suggesting that increased model-free control is beneficial to overall performance. Why might this be? To perform optimally, participants continually have to update the estimates of second-stage outcomes (aliens) according to the randomly drifting outcome probabilities. It turns out that this stochasticity imposes a low ceiling on achievable performance on this task, such that the theoretical winnings do not differ between pure model-based, model-free and random agents (Akam et al., 2015; Kool et al., 2016). Over the last decade, various versions of the task have been introduced and concern has been raised about the interpretation of the findings. Although it has been shown that model-free behavior can be confused for model-based behavior (Akam et al., 2015) and model-based behavior can be confused for model-free behavior (Feher da Silva & Hare, 2020), we emphasize that our effects are within-subject changes, so any biases would affect both sessions equally. We also opted to use this particular design as a continuation of a previous study by Friedel et al., (2014). However, we cannot rule out that hunger could decrease attention to the instructions, which can enhance the model-free behavior.

A previous study by Friedel et al., (2014) hinted towards a relationship between model-based control and metabolic need. Friedel et al., indexed the metabolic need of an individual as the change in valuation for food rewards in a separate devaluation paradigm. They observed that people who exhibited greater devaluation also showed more model-based control. In contrast, our study asks whether changes in metabolic needs have generalized effects on cognition, beyond the specific reward type that changes in value. By testing the same individuals twice on this sequential decision-making task (once in high metabolic need and once in low metabolic need), we were able to directly examine the causal effect of metabolic need on the balance between model-based and model-free control in the same individual, and correlate this with overall performance. Crucially, our observations are complementary to those of Friedel et al., (2014). Although metabolic changes did not affect model-based behavior for *monetary* outcomes, they may still affect the value of food rewards. Indeed, the value of a particular reward increases when humans or animals are deprived of it (Aw et al., 2009; Epstein et al., 2003; Pompilio, Kacelnik, & Behmer, 2006; Siep et al., 2009). Instead, the increased learning we observed must reflect a more general change in cognition, perhaps associated with heightened motivation.

How might such general effects arise? One mechanism may be that food deprivation acts as a mild stressor (Deroche et al., 1995). Stress itself may impair model-based control (Otto, Raio, Chiang, Phelps, & Daw, 2013) and enhances reliance on model-free strategies, particularly for negative outcomes (Park, Lee, & Chey, 2017). This may arise through the action of various metabolic hormones, as well as hypothalamic inputs to the ventral prefrontal cortex. It is worth noting that the results are incompatible with hunger simply disrupting attention, which would be expected to *decrease* learning rates and performance.

Metabolic hormones mostly act on primitive, subcortical areas (Elmquist et al., 1998; Zigman et al., 2006), including the basal ganglia. These areas play an important role in model-free control (Gläscher et al., 2010; Daw et al., 2011; Lee et al., 2014; Deserno et al., 2015; Smittenaar et al., 2013), and are modulated by the current metabolic state (Cone et al., 2016; Aitken et al., 2016; Papageorgiou et al., 2016; Hommel et al., 2006; Abizaid et al., 2006). This link may support our behavioral and modeled findings that hunger increased model-free control, without affecting model-based decision-making.

Our findings may be directly relevant to populations with dysregulation of hunger. Obese individuals with eating disorders, but not obese individuals without eating disorders, exhibit increased model-free behavior in this same task (Voon et al., 2015). Our study provides a crucial causal link, that within individual participants from an unselected population, hunger increases model-free behavior. We only had a small range of BMI in our sample and thus are not powered to detect the effects of BMI on effects of hunger on this task. However, there was a suggestion that people with a higher BMI have stronger hunger effects (see Supplementary Material). Future studies may include individuals with a wider range of BMI and individuals with eating disorders, such as anorexia nervosa and binge eating disorder. Given that cognitive control is enhanced in anorexia nervosa and reduced in binge eating disorder (Higgs et al., 2017), the effects of hunger on these populations might differ from the individuals in this study who have a normal weight and normal eating habits.

To conclude, we found that increased metabolic need enhanced reflexive model-free control of behavior, without affecting deliberate model-based control. Moreover, relying on a reflexive model-free system does not necessarily make hungry people maladaptive, when the deliberate model-based system remains unaffected.

## Electronic supplementary material

Below is the link to the electronic supplementary material.

(PDF 558 KB)

## References

[CR1] Abizaid A, Liu Z-W, Andrews ZB, Shanabrough M, Borok E, Elsworth JD, Roth RH, Sleeman MW, Picciotto MR, Tschop MH, Gao X-B, Horvath TL (2006). Ghrelin modulates the activity and synaptic input organization of midbrain dopamine neurons while promoting appetite. The Journal of Clinical Investigation.

[CR2] Aitken TJ, Greenfield VY, Wassum KM (2016). Nucleus accumbens core dopamine signaling tracks the need-based motivational value of food-paired cues. Journal of Neurochemistry.

[CR3] Akam T, Costa R, Dayan P (2015). Simple plans or sophisticated habits? State, transition and learning interactions in the two-step task. PLOS Computational Biology.

[CR4] Aw JM, Holbrook R, Burt de Perera T, Kacelnik A (2009). State-dependent valuation learning in fish: Banded tetras prefer stimuli associated with greater past deprivation. Behavioural Processes.

[CR5] Bartholdy S, Cheng J, Schmidt U, Campbell IC, O’Daly OG (2016). Task-based and questionnaire measures of inhibitory control are differentially affected by acute food restriction and by motivationally salient food stimuli in healthy adults. Frontiers in Psychology.

[CR6] Bond A, Lader M (1974). The use of analogue scales in rating subjective feelings. British Journal of Medical Psychology.

[CR7] Briers B, Pandelaere M, Dewitte S, Warlop L (2006). Hungry for money. Psychological Science.

[CR8] Compan V, Walsh BT, Kaye W, Geliebter A (2015). How does the brain implement adaptive decision making to eat?. Journal of Neuroscience.

[CR9] Cone JJ, Fortin SM, McHenry JA, Stuber GD, McCutcheon JE, Roitman MF (2016). Physiological state gates acquisition and expression of mesolimbic reward prediction signals. Proceedings of the National Academy of Sciences of the United States of America.

[CR10] Daw ND, Gershman SJ, Seymour B, Dayan P, Dolan RJ (2011). Model-based influences on humans’ choices and striatal prediction errors. Neuron.

[CR11] de Ridder D, Kroese F, Adriaanse M, Evers C (2014). Always gamble on an empty stomach: hunger is associated with advantageous decision making. PLoS ONE.

[CR12] Deroche V, Marinelli M, Maccari S, Le Moal M, Simon H, Piazza PV (1995). Stress-induced sensitization and glucocorticoids. I. Sensitization of dopamine-dependent locomotor effects of amphetamine and morphine depends on stress-induced corticosterone secretion. Journal of Neuroscience.

[CR13] Deserno L, Huys QJ, Boehme R, Buchert R, Heinze HJ, Grace AA, Dolan RJ, Heinz A, Schlagenhauf F (2015). Ventral striatal dopamine reflects behavioral and neural signatures of model-based control during sequential decision making. Proceedings of the National Academy of Sciences of the United States of America.

[CR14] Dickinson A, Balleine BW (1994). Motivational control of goal-directed action. Animal Learning and Behavior.

[CR15] Dickinson AD (1985). Actions and habits: the development of behavioural autonomy. Philosophical Transactions of the Royal Society of London B, Biological Sciences.

[CR16] Elmquist JK, Bjørbæk C, Ahima RS, Flier JS, Saper CB (1998). Distributions of leptin receptor mRNA isoforms in the rat brain. Journal of Comparative Neurology.

[CR17] Epstein LH, Truesdale R, Wojcik A, Paluch RA, Raynor HA (2003). Effects of deprivation on hedonics and reinforcing value of food. Physiology and Behavior.

[CR18] Feher da Silva C, Hare T (2020). Humans primarily use model-based inference in the two-stage task. Nature Human Behaviour.

[CR19] Figlewicz DP, MacDonald Naleid A, Sipols AJ (2007). Modulation of food reward by adiposity signals. Physiology and Behavior.

[CR20] Flint A, Raben A, Blundell JE, Astrup A (2000). Reproducibility, power and validity of visual analogue scales in assessment of appetite sensations in single test meal studies. International Journal of Obesity.

[CR21] Friedel E, Koch SP, Wendt J, Heinz A, Deserno L, Schlagenhauf F (2014). Devaluation and sequential decisions: linking goal-directed and model-based behavior. Frontiers in Human Neuroscience.

[CR22] Gläscher J, Daw N, Dayan P, O’Doherty JP (2010). States versus rewards: Dissociable neural prediction error signals underlying model-based and model-free reinforcement learning. Neuron.

[CR23] Glimcher PW, Fehr E (2013). Neuroeconomics: Decision Making and the Brain: Second Edition.

[CR24] Grosskurth ED, Bach DR, Economides M, Huys QJM, Holper L (2019). No substantial change in the balance between model-free and model-based control via training on the two-step task. PLoS Computational Biology.

[CR25] Guitart-Masip M, Fuentemilla L, Bach DR, Huys QJM, Dayan P, Dolan RJ, Duzel E (2011). Action dominates valence in anticipatory representations in the human striatum and dopaminergic midbrain. The Journal of Neuroscience : The Official Journal of the Society for Neuroscience.

[CR26] Haase L, Cerf-Ducastel B, Murphy C (2009). Cortical activation in response to pure taste stimuli during the physiological states of hunger and satiety. NeuroImage.

[CR27] Hassenstab JJ, Sweet LH, Del Parigi A, McCaffery JM, Haley AP, Demos KE, Cohen RA, Wing RR (2012). Cortical thickness of the cognitive control network in obesity and successful weight loss maintenance: A preliminary MRI study. Psychiatry Research - Neuroimaging.

[CR28] Higgs S, Spetter MS, Thomas JM, Rotshtein P, Lee M, Hallschmid M, Dourish CT (2017). Interactions between metabolic, reward and cognitive processes in appetite control: Implications for novel weight management therapies. Journal of Psychopharmacology.

[CR29] Hommel JD, Trinko R, Sears RM, Georgescu D, Liu ZW, Gao XB, Thurmon JJ, Marinelli M, DiLeone RJ (2006). Leptin receptor signaling in midbrain dopamine neurons regulates feeding. Neuron.

[CR30] Kirk J, Logue A (1997). Effects of deprivation level on humans’ Self-Control for food reinforcers. Appetite.

[CR31] Kool W, Cushman FA, Gershman SJ (2016). When does model-based control pay off?. PLOS Computational Biology.

[CR32] Lee SW, Shimojo S, O’Doherty JP (2014). Neural computations underlying arbitration between model-based and model-free learning. Neuron.

[CR33] Levy DJ, Thavikulwat AC, Glimcher PW (2013). State dependent valuation: the effect of deprivation on risk preferences. PLoS ONE.

[CR34] MacKay DJC (2003). Information theory, inference, and learning algorithms, Vol. 13.

[CR35] Malik S, McGlone F, Bedrossian D, Dagher A (2008). Ghrelin modulates brain activity in areas that control appetitive behavior. Cell Metabolism.

[CR36] Otto AR, Raio CM, Chiang A, Phelps EA, Daw ND (2013). Working-memory capacity protects model-based learning from stress. Proceedings of the National Academy of Sciences.

[CR37] Palminteri S, Lefebvre G, Kilford EJ, Blakemore SJ (2017). Confirmation bias in human reinforcement learning: Evidence from counterfactual feedback processing. PLoS Computational Biology.

[CR38] Papageorgiou GK, Baudonnat M, Cucca F, Walton ME (2016). Mesolimbic dopamine encodes prediction errors in a state-dependent manner. Cell Reports.

[CR39] Park H, Lee D, Chey J (2017). Stress enhances model-free reinforcement learning only after negative outcome. PLoS ONE.

[CR40] Pompilio L, Kacelnik A, Behmer ST (2006). State-dependent learned valuation drives choice in an invertebrate. Science.

[CR41] Radenbach C, Reiter AM, Engert V, Sjoerds Z, Villringer A, Heinze HJ, Deserno L, Schlagenhauf F (2015). The interaction of acute and chronic stress impairs model-based behavioral control. Psychoneuroendocrinology.

[CR42] Rummery, G.A., & Niranjan, M. (1994). On-Line Q-Learning Using connectionist systems. Technical report, Cambridge University Engineering Department.

[CR43] Saper, C.B., Chou, T.C., & Elmquist, J.K. (2002). The need to feed: Homeostatic and hedonic control of eating. 10.1016/s0896-6273(02)00969-8 12383777

[CR44] Shabat-Simon M, Shuster A, Sela T, Levy DJ (2018). Objective physiological measurements but not subjective reports moderate the effect of hunger on choice behavior. Frontiers in Psychology.

[CR45] Shahar, N., Moran, R., Hauser, T.U., Kievit, R.A., McNamee, D., Moutoussis, M., ..., Dolan, R.J. (2019). Credit assignment to state-independent task representations and its relationship with model-based decision making, (Vol. 116 pp. 15871– 15876). 10.1073/pnas.1821647116 PMC6689934 31320592

[CR46] Siep N, Roefs A, Roebroeck A, Havermans R, Bonte ML, Jansen A (2009). Hunger is the best spice: An fMRI study of the effects of attention, hunger and calorie content on food reward processing in the amygdala and orbitofrontal cortex. Behavioural Brain Research.

[CR47] Simon JJ, Wetzel A, Sinno MH, Skunde M, Bendszus M, Preissl H, Enck P, Herzog W, Friederich H-C (2017). Integration of homeostatic signaling and food reward processing in the human brain Joe. JCI Insight.

[CR48] Skrynka J, Vincent BT (2019). Hunger increases delay discounting of food and non-food rewards. Psychonomic Bulletin and Review.

[CR49] Smittenaar P, FitzGerald THB, Romei V, Wright ND, Dolan RJ (2013). Disruption of dorsolateral prefrontal cortex decreases model-based in favor of model-free control in humans. Neuron.

[CR50] Symmonds M, Emmanuel JJ, Drew ME, Batterham RL, Dolan RJ (2010). Metabolic state alters economic decision making under risk in humans. PloS ONE.

[CR51] van Swieten, M.M.H. (2020). Physiological modulation of learning and decision-making. PhD thesis, University of Oxford.

[CR52] van Swieten, M.M.H., Manohar, S.G., & Bogacz, R. (2021). Effects of hunger on model-based and model-free decision-making.

[CR53] Voon V, Derbyshire K, Rück C, Irvine MA, Worbe Y, Enander J, Schreiber LR, Gillan C, Fineberg NA, Sahakian BJ, Robbins TW, Harrison NA, Wood J, Daw ND, Dayan P, Grant JE, Bullmore ET (2015). Disorders of compulsivity: a common bias towards learning habits. Molecular Psychiatry.

[CR54] Wilson RC, Collins AG (2019). Ten simple rules for the computational modeling of behavioral data. eLife.

[CR55] Wunderlich K, Smittenaar P, Dolan RJ (2012). Dopamine enhances Model-Based over Model-Free choice behavior. Neuron.

[CR56] Zigman JM, Jones JE, Lee CE, Saper CB, Elmquist JK (2006). Expression of ghrelin receptor mRNA in the rat and the mouse brain. The Journal of Comparative Neurology.

