# Supplementary material for: Hunger improves reinforcement-driven but not planned action
Source: Cogn Affect Behav Neurosci. 2021 Oct 15;21(6):1196–206. doi: 10.3758/s13415-021-00921-w (PMC8563670; doi:10.3758/s13415-021-00921-w)
Supplement: Supplementary file 1 — (PDF 558 KB) [file 13415_2021_921_MOESM1_ESM.pdf]

Supplemental material

Other reinforcement-driven behaviors were not affected

In addition to the stay-switch measure reported in Fig.2, there are two other measures of simple reinforcement learning in this task that are independent of previous state transitions. The first measure is stay behavior for second-stage choices, which is indicative of adaptive behavior driven by reinforcements. The win-stay and lose-stay probabilities for second-stages were calculated for the two available stimuli together, but separately for the two planets, and divided by the number of wins or losses to give the percentage of stay behavior for each participant. For this analysis, we assumed that the same state did not have to be visited twice in a row, because this would make the analysis dependent on the first-stage choices. Consequently, we assumed that participants were able to keep an average of the total expected value for a stimulus in their working memory. Participants were more likely to repeat their previous choice after receiving a reward (main effect of reward [$F_{1,31} = 124.50$, $p < 0.0001$, $\eta_p^2 = 0.80$]). Food deprivation did not alter overall stay behavior (main effect of food deprivation [$F_{1,31} < 1$]) or outcome-specific stay behavior (interaction effect of food deprivation and reward [$F_{1,31} < 1$]; Fig. S1A).

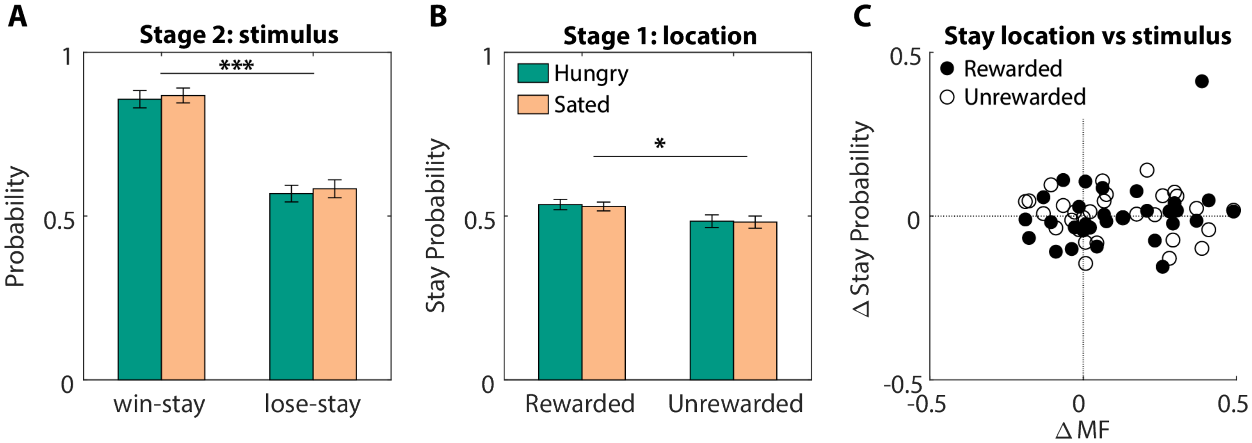

Figure S1: **Hunger did not alter other types of reinforcement learning. A)** Repetition of second-stage choices was enhanced following rewarded trials and was not altered by food deprivation. **B)** Repetition of first-stage actions was enhanced following rewarded trials, regardless of stimulus identity or the level of food deprivation. **C)** There was no correlation between first-stage action repetition and the model-free (MF) values of first-stage choices. Action repetition did not contribute to model-free control of stage 1 choices. Error bars represent SEM. * $p < 0.05$, *** $p < 0.001$.

The second measure is stay-switch behavior for outcome-irrelevant information (Shahar et al., 2019). Participants may associate actions (i.e. choosing left or right), rather than stimulus identity, with reward outcomes and repeat an action after receiving a reward on

the previous trial for that action. Given that only the stimulus identity, not the stimulus location, has predictive effects, this type of behavior could be seen as a measure of aberrant incidental learning, in which a value is assigned to an action that was not inherently rewarding. Participants repeated the same action more often after receiving a reward (main effect of reward [$F_{1,31} = 6.34$, $p = 0.017$, $\eta_p^2 = 0.17$]). Food deprivation did not alter overall stay behavior (main effect of food deprivation [$F_{1,31} < 1$]) or outcome-specific stay behavior (interaction effect of food deprivation and reward [$F_{1,31} < 1$]; Fig. S1B). The significant effect of reward on action repetition did not affect the contribution of the model-free system to first-stage choices (Fig. S1C). Together, these analyses show that food deprivation selectively affected model-free control of first-stage choices, but not all types of reinforcement-driven learning.

Computational modelling

We optimized the model parameters by minimizing the negative log-likelihood (LL) of the data, given different parameter settings, using MATLAB's `fminunc` function to obtain unconstrained parameter estimates:

$$\mathrm{LL} = \log(P(\mathrm{data}|\mathrm{model})) \tag{1}$$

Parameter transformations

The unconstrained Gaussian distributed parameter estimates $x_i \sim \mathcal{N}(\mu_x, \sigma_x)$, with a population mean of μ_x and a standard deviation of σ_x, were transformed into bounded model parameters using logistic/exponential transformations to ensure that the model parameters were biologically plausible and interpretable. This transformation is justified by the premise that each individual subject (with a parameter value x_i) is randomly drawn from a population of subjects with normally distributed parameters (with population mean μ_x and a standard deviation of σ_x) (Daw et al., 2011; Wunderlich et al., 2012). This is important for our analysis because normally distributed parameters permit the use of parametric tests to identify differences between conditions. We denote the model parameters by Greek letters and the Gaussian scaled parameter estimates by their respective Latin letters.

The relationship between the [0,1]-bounded model parameter α and the Gaussian parameter estimate a is given by a logistic function:

$$\alpha = 1/(1 + \exp(-a)). \tag{2}$$

The relationships between the $[0, \infty)$-bounded (logarithmically scaled) model parameters β

and π and the Gaussian parameter estimates b and p are given by the exponential function:

$$\beta = \exp(b), \tag{3}$$

$$\pi = \exp(p). \tag{4}$$

Parameter recovery

To validate the parameter estimates generated by the fitting procedure, we conducted a parameter recovery analysis. For each parameter, we generated samples from the marginalized posterior distribution of the fit to get realistic parameters that could describe choice behavior. The generated parameters were uncorrelated ($|R| < 0.3$), allowing for testing whether the fitting procedure introduced any confounding factors. Using these parameters, we simulated choice behavior for 32 virtual subjects following the task design described in the method section. We then used a hierarchical model fitting procedure to estimate parameters for the simulated data ("Recovered parameters"). We assessed the quality of the parameter recovery by comparing the true parameters used to simulate data with the recovered parameters. We calculated the Pearson correlation between all pairs of recovered parameters to test whether the fitting procedure introduced spurious correlations. A strong correlation between the true and recovered parameters indicates a good recovery of the parameters and reliable model fitting results. All parameters were well recovered ($0.65 < R < 0.95$) and the model fitting procedure did not introduce spurious correlations between the other parameters ($|R| < 0.4$; Fig. S2 & S3).

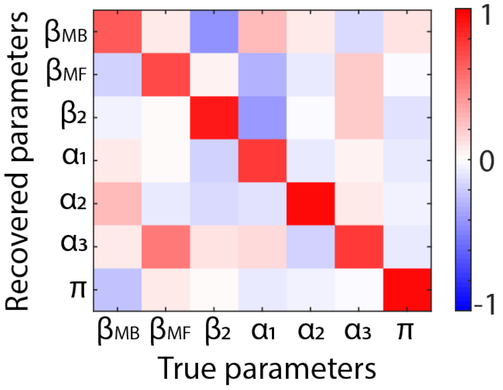

Figure S2: **Confusion matrix.** Correlation matrix of the free parameters used to generate the simulated data ('True parameters') and the obtained parameters by applying the parameter estimation procedure on the simulated data ('Recovered parameters'). Bright red values indicate a strong correlation between the true and recovered parameter value and therefore a good parameter recovery.

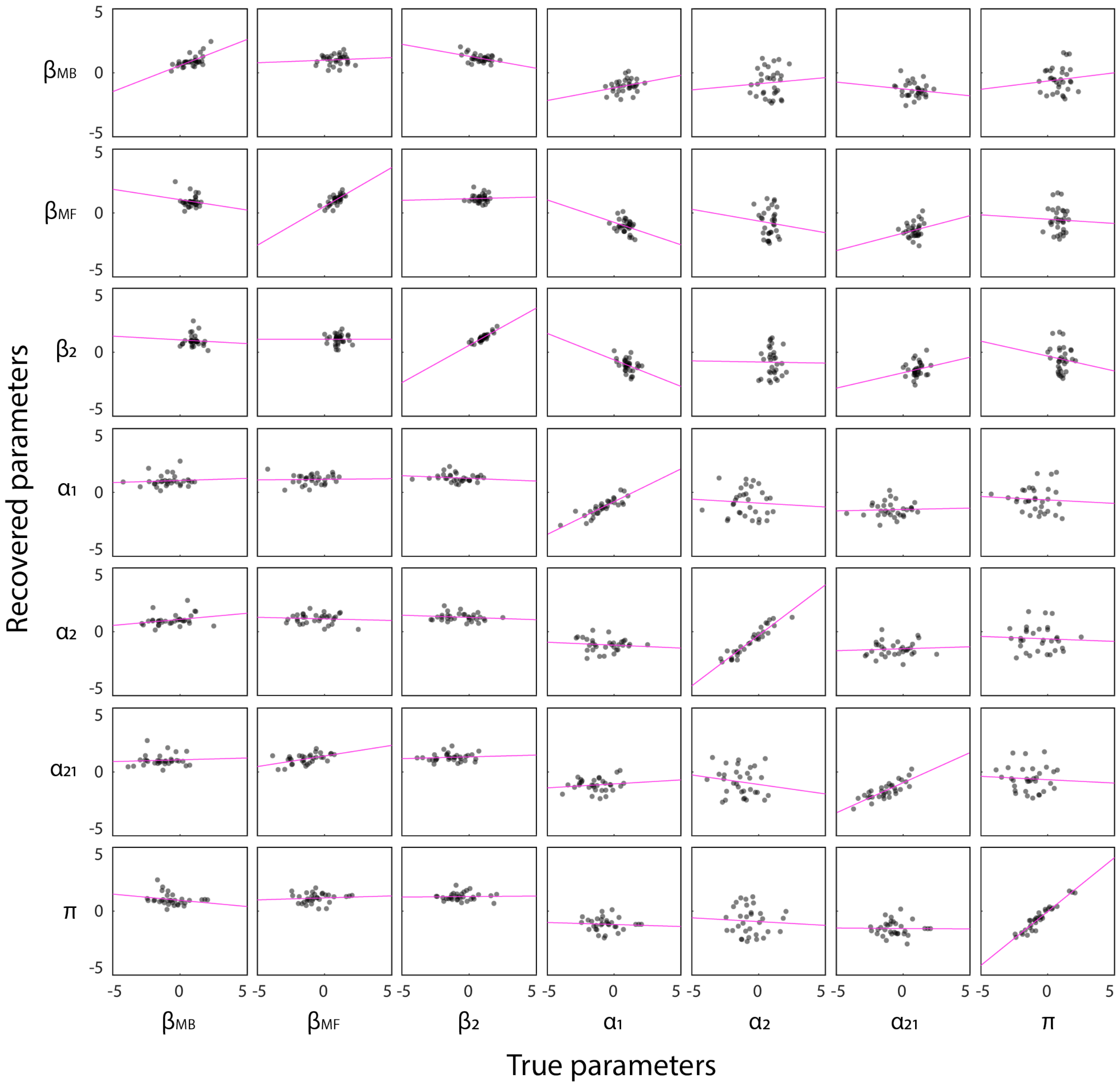

Figure S3: **Parameter recovery.** Correlations of the free parameters used to generate the simulated data ('True parameters') and the obtained parameters by applying the parameter estimation procedure on the simulated data ('Recovered parameters').

Surrogate data

To confirm that our model can capture our key behavioral findings, we generated data for 32 virtual subjects on this task using the individual best-fitting parameters (Table 1) and the same trajectories of reward probabilities as experienced by the participant. Each simulation was repeated 30 times to obtain an average. These data were then subjected to a stay-switch analysis. We found an identical pattern of effects in these generated data as observed

empirically in our participants (Fig. 4C).

	$\beta_{\mathbf{MB}}$	$\beta_{\mathbf{MF}}$	β_2	α_1	α_2	α_{21}	π
**Sated**							
25th percentile	1.43	1.40	2.13	0.15	0.30	0.08	0.30
Median	2.25	2.10	3.64	0.23	0.49	0.14	0.67
75th percentile	4.22	2.96	5.15	0.30	0.64	0.23	1.38
**Food deprived**							
25th percentile	1.65	1.86	2.37	0.34	0.11	0.17	0.27
Median	3.09	2.35	4.23	0.45	0.40	0.22	0.72
75th percentile	7.03	3.01	4.93	0.54	0.58	0.26	1.13

Table 1: **Best-fitting model parameters estimates.** Separately shown for the sated and food deprived condition as median and quartiles across participants.

Main effects for individual participants

We visualized the main effects for individual participants in Fig. S4. More points are below the diagonal in panel A, consistent with the effect of hunger on model-free behavior, while the points in panel B are equally distributed on both sides of the diagonal, consistent with the lack of an effect of hunger on model-based behavior. There was no relation between model-based and model-free indices in either session (panel C & D in Fig. S4).

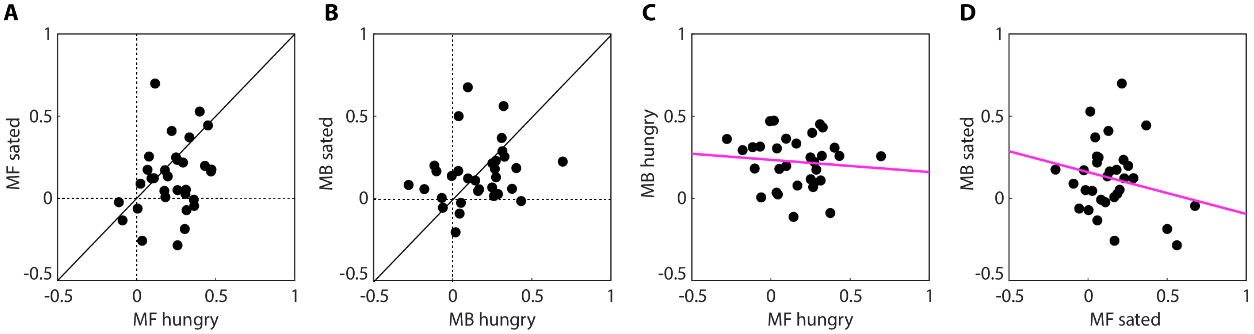

Figure S4: **Main effects for individual participants.** Each individual participant is represented by a single point. **A)** Individuals became more model-free (MF) when hungry, represented by a greater number of points below the diagonal. **B)** There is no effect of hunger on model-based (MB) behavior, represented by the equal distribution of points on either side of the diagonal. (**C & D**) There is no relation between MB and MF indices for either session.

Note that our sample size and design was not intended to examine between subjects effects. However, we wanted to rule out that age might drive some of the effects we see. Age was included in the ANOVA as a covariate, and there were no significant effects or interactions involving age.

Previous studies have reported effects of BMI on model-based decision-making (Voon et al., 2015). Hence, we asked whether we could see this effect in our sample. Including BMI as a covariate revealed several significant three-way interactions with BMI. Since this was a four-way ANOVA, which are notoriously difficult to interpret, we performed a median split on BMI. Heavier participants showed a strong effect of hunger on model-free learning ($F = 8.61$, $p = 0.010$), the same as reported at the group level. Lighter participants, in contrast, did not ($F = 0.57$, $p = 0.461$). Unexpectedly, these participants showed an effect of hunger on transition ($F = 10.07$, $p = 0.006$), which we can only explain as an anomaly of being underpowered for this sub-analysis.

References

Daw, N. D., Gershman, S. J., Seymour, B., Dayan, P., and Dolan, R. J. (2011). Model-based influences on humans' choices and striatal prediction errors. *Neuron*, 69(6):1204–1215.

Shahar, N., Moran, R., Hauser, T. U., Kievit, R. A., McNamee, D., Moutoussis, M., Nspn, C., and Dolan, R. J. (2019). Credit assignment to state-independent task representations and its relationship with model-based decision making. *Proceedings of the National Academy of Sciences of the United States of America*, 116(32):15871–15876.

Voon, V., Derbyshire, K., Rück, C., Irvine, M. A., Worbe, Y., Enander, J., Schreiber, L. R., Gillan, C., Fineberg, N. A., Sahakian, B. J., Robbins, T. W., Harrison, N. A., Wood, J., Daw, N. D., Dayan, P., Grant, J. E., and Bullmore, E. T. (2015). Disorders of compulsivity: A common bias towards learning habits. *Molecular Psychiatry*, 20(3):345–352.

Wunderlich, K., Smittenaar, P., and Dolan, R. J. (2012). Dopamine Enhances Model-Based over Model-Free Choice Behavior. *Neuron*, 75(3):418–424.
